# The value of perioperative nutritional risk stratification in the prevention of surgical site infection: precision application from GLIM/PG-SGA to PNI, CONUT, and GNRI

**DOI:** 10.3389/fnut.2026.1837138

**Published:** 2026-07-24

**Authors:** Qiang Hu, Yuanshui Sun

**Affiliations:** Tongde Hospital of Zhejiang Province Affiliated to Zhejiang Chinese Medical University (College of Integrated Traditional Chinese and Western Medicine Clinical Medicine), Hangzhou, Zhejiang, China

**Keywords:** CONUT, geriatric nutritional risk index, GLIM, nutritional risk stratification, perioperative malnutrition, PG-SGA, prognostic nutritional index, surgical site infection

## Abstract

Surgical site infection (SSI) remains a major postoperative complication despite advances in perioperative prophylaxis and standardized care. Increasing evidence suggests that perioperative malnutrition is a host-related condition linking systemic inflammation, impaired immune recovery, low muscle mass, delayed wound healing, and increased susceptibility to infection. This narrative review summarizes the biological relationship between perioperative malnutrition and SSI, critically compares the roles of GLIM, PG-SGA, PNI, CONUT, and GNRI, and distinguishes SSI-specific findings from evidence based on broader postoperative outcomes. GLIM and PG-SGA are most useful for defining clinically meaningful malnutrition phenotypes, whereas PNI, CONUT, and GNRI support rapid and objective risk quantification. Because these tools capture different dimensions of nutritional vulnerability, we propose a complementary, stepwise pathway integrating early screening, phenotype-based diagnosis, secondary risk stratification, targeted intervention, and postoperative reassessment. This framework should be regarded as a hypothesis-generating clinical model that requires prospective validation before it can be adopted as a standardized SSI-prevention algorithm.

## Highlights

- Perioperative malnutrition is a host-related driver of SSI susceptibility.
- GLIM and PG-SGA identify malnutrition phenotypes with clinical relevance.
- PNI, CONUT, and GNRI support rapid and objective SSI risk quantification.
- Nutritional tools may be most useful when applied in a complementary stepwise pathway.
- Precision nutritional stratification may improve targeted perioperative SSI prevention.

## 1 Introduction

Surgical site infection (SSI) is one of the most common and clinically significant infectious complications in the perioperative period. It can prolong hospital stay, increase readmission and reoperation rates, raise healthcare costs, and delay patient recovery (1, 2). At present, SSI prevention has evolved into a relatively mature technical framework, mainly including perioperative antibiotic prophylaxis, alcohol-based skin antisepsis, perioperative glycemic control, maintenance of normothermia, and other process optimization measures during the perioperative period (3). Nevertheless, in clinical practice, patients undergoing similar operations and receiving standard preventive measures may still show markedly different risks of infection. In recent years, this phenomenon has prompted renewed attention to the role of host-related factors in the development of SSI. Notably, the updated 2025 ESPEN guideline has incorporated nutritional management into the overall perioperative treatment framework and emphasized that nutritional intervention should be initiated as early as possible once nutritional risk is identified (4). Therefore, whether perioperative nutritional status is an important and modifiable factor influencing the occurrence of SSI deserves further in-depth investigation.

Previous studies on perioperative nutrition have mainly focused on the impact of nutritional support on postoperative outcomes, with particular attention to the efficacy of immunonutrition, the optimal timing of early enteral nutrition, and nutritional management strategies within enhanced recovery after surgery (ERAS) programs (5–7). Recent reviews have expanded this field. Noorian et al. summarized perioperative nutritional assessment and the route, timing, and duration of nutritional support in gastrointestinal surgery (8). Gazouli et al. (9) reviewed perioperative nutritional assessment and management for patients undergoing gastrointestinal surgery. Wobith et al. (10) focused on nutritional prehabilitation in abdominal surgery, while the updated Cochrane review by Sowerbutts et al. (11) evaluated preoperative nutritional therapy in gastrointestinal surgery.

These reviews are clinically valuable, but their primary emphasis is nutritional support, prehabilitation, or overall postoperative outcomes. They do not systematically distinguish superficial incisional, deep incisional, and organ/space SSI from broader infectious or overall complications. They also do not integrate GLIM/PG-SGA with PNI, CONUT, and GNRI into an SSI-oriented stepwise pathway. This unresolved gap provides the rationale for the present review.

Comparative studies suggest that different nutritional tools may not identify the same risk state. PG-SGA and GLIM appear more suitable for frontline identification and clinical phenotyping, whereas CONUT and GNRI may serve as objective supplements for subsequent quantification (12). The agreement between GLIM and PG-SGA may also depend on the screening approach and workflow design, suggesting that these tools are complementary rather than interchangeable (13). PNI has been used to predict SSI risk in selected surgical populations; however, the respective applications and synergistic value of PNI, CONUT, GNRI, and GLIM/PG-SGA still lack an SSI-oriented synthesis (14).

Prospective multicenter studies have shown that poor preoperative nutritional status is associated with postoperative infections in patients with gastrointestinal malignancies. Higher NRS-2002 scores in gastric cancer and PG-SGA grade B/C in colorectal cancer have both been linked to increased postoperative infection risk (15). The GLIM consensus also provides a standardized framework for diagnosing adult malnutrition (16). Against this background, this review aims to: (1) summarize the biological link between perioperative malnutrition and SSI; (2) critically compare GLIM, PG-SGA, PNI, CONUT, and GNRI, with explicit separation of SSI-specific evidence from evidence based on overall complications; and (3) propose a practical, hypothesis-generating pathway for nutritional risk stratification and targeted perioperative intervention.

## 2 Methods

This article is a structured narrative review rather than a systematic review or meta-analysis. To improve transparency, relevant literature was identified through searches of PubMed, Embase, Web of Science and Cochrane Library, supplemented by manual screening of the reference lists of key guidelines, systematic reviews, meta-analyses, and clinically relevant original studies. The search covered publications available up to 1 Mar 2026. Search terms were combined around four domains: surgery and perioperative care; SSI or postoperative infection; malnutrition or nutritional risk; and specific tools, including GLIM, PG-SGA, PNI, CONUT, GNRI, NRS-2002, sarcopenia, frailty, ERAS, and nutritional prehabilitation.

We prioritized adult human studies that evaluated SSI, postoperative infectious complications, or clinically relevant postoperative outcomes in surgical populations. Guidelines, systematic reviews, meta-analyses, prospective cohorts, retrospective cohorts, and selected randomized trials of perioperative nutritional intervention were considered. Studies focused on non-surgical populations, case reports, conference abstracts without full text, and duplicate reports were not used as core evidence. When SSI-specific studies were limited, evidence based on overall complications was retained as supportive evidence and is explicitly labeled as indirect. Because the purpose of this review was interpretive synthesis rather than pooled effect estimation, no formal risk-of-bias tool or quantitative meta-analysis was applied.

## 3 Biological link between perioperative malnutrition and SSI

Perioperative malnutrition is not limited to inadequate caloric intake. It is a host state in which reduced nutritional substrates, systemic inflammation, and impaired immune function interact. Protein and amino acid deficiency can weaken lymphocyte proliferation, cytokine release, antibody production, macrophage phagocytosis, and pathogen killing (17–19). Surgical trauma adds a second insult by inducing inflammatory activation followed by compensatory immune suppression. Patients who are already malnourished may therefore experience slower postoperative immune recovery and persistent lymphopenia (17, 20).

The role of malnutrition in promoting SSI is also reflected in its persistent impact on the entire wound-healing process. Normal wound repair generally consists of three overlapping stages: the inflammatory phase, the proliferative phase, and the remodeling phase. During the inflammatory phase, immune cells must be rapidly recruited to clear pathogens and necrotic tissue. Malnutrition weakens this process, resulting in a less efficient and prolonged local inflammatory response. During the proliferative phase, fibroblast migration, collagen deposition, angiogenesis, and re-epithelialization all depend on adequate protein, energy, and multiple micronutrients; nutritional deficiency can therefore lead to insufficient granulation tissue formation, reduced collagen synthesis, and delayed tissue regeneration. During the remodeling phase, the extracellular matrix continues to be reconstructed and tensile strength is gradually restored. If nutritional support has been inadequate during the earlier stages, scar maturation is delayed, tissue strength recovers poorly, and the risk of wound dehiscence and secondary infection may increase (21, 22). Previous studies have shown that protein deficiency adversely affects nearly the entire process of immune defense and tissue repair, whereas carbohydrates, vitamins, and trace elements are involved in key processes such as leukocyte function, fibroblast migration, collagen synthesis, and angiogenesis (21). Among these factors, collagen, as one of the most critical extracellular matrix components in wound healing, is involved throughout the inflammatory, proliferative, and remodeling phases. Once its synthesis, degradation, and remodeling become unbalanced, persistent wound healing failure and ongoing infection may result (22).

**Figure 1:**
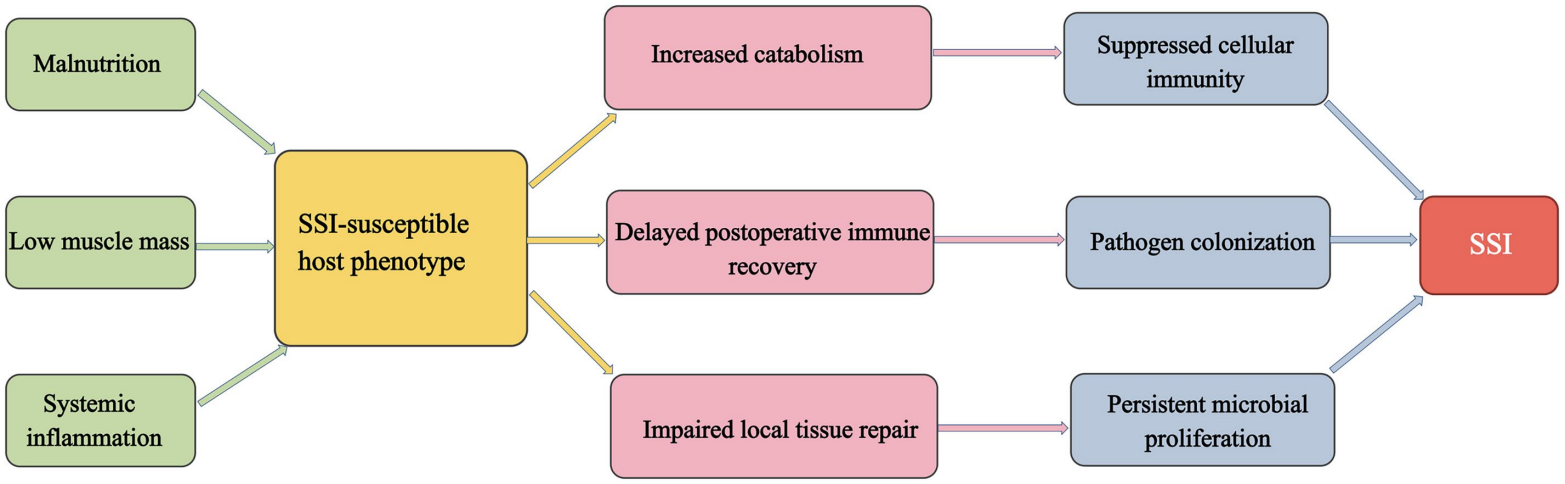
Mechanistic pathway by which perioperative malnutrition promotes SSI.

Taken together, malnutrition, low muscle mass, and systemic inflammation create an SSI-susceptible host phenotype. Surgical stress intensifies catabolism and suppresses cellular immunity, while limited nutritional reserve impairs early immune reconstruction and local tissue repair. This combination favors pathogen colonization, microbial proliferation, delayed wound healing, and secondary infection (20–23) (Figure 1). In patients with cancer, the risk is amplified by cachexia, hypercatabolism, reduced intake, and inflammatory activation (24, 25).

Several clinical phenotypes further amplify this risk. Cancer and older age are frequently accompanied by hypercatabolism, immunosenescence, and micronutrient deficiencies. Sarcopenia and frailty are associated with worse postoperative outcomes, and diabetes or perioperative hyperglycemia can impair neutrophil migration, phagocytosis, oxidative burst, and pathogen killing (26–31). These factors should be interpreted as interacting components of host vulnerability rather than as isolated predictors.

## 4 Conceptual framework of perioperative nutritional risk stratification

Perioperative nutritional management first requires clarification of several related but conceptually distinct terms. Nutritional screening refers to the use of simple and rapid tools, either after hospital admission or in the early preoperative period, to initially identify patients who may be at nutritional risk; its purpose is to promptly detect individuals who require further intervention. Nutritional assessment, by contrast, is performed after a positive screening result and involves a more comprehensive evaluation of the patient’s nutritional status by integrating information on medical history, dietary intake, symptom burden, physical examination, anthropometric measurements, functional status, and body composition. The diagnosis of malnutrition is the formal determination of whether malnutrition is present and, if so, its severity, based on specific criteria. Risk stratification goes a step further by integrating factors such as age, inflammatory burden, degree of surgical trauma, underlying diseases, and physiological reserve in order to assess the risk of postoperative complications, especially SSI. Finally, treatment targeting should be based on the results of this stratification to determine whether preoperative prehabilitation, intensified nutritional support, postponement of elective surgery, or closer postoperative infection monitoring is warranted (32–35). In other words, screening answers the question of “who may have a problem,” assessment and diagnosis address “what exactly the problem is and how severe it is,” whereas risk stratification and treatment targeting focus on “who is more likely to develop adverse outcomes and what intervention strategy should be adopted.”

Based on this rationale, perioperative nutritional risk stratification is better approached using a two-step or stepwise framework. The first step is to identify patients with malnutrition or high nutritional risk. In oncologic surgery populations, PG-SGA is more suitable as a frontline identification tool; in contrast, a GLIM-centered pathway emphasizes first identifying high-risk patients through validated screening tools and then proceeding to a more detailed diagnostic assessment, thereby allowing characterization of both the malnutrition phenotype and its underlying etiology (36, 37). The second step, after confirming the presence of nutritional abnormalities, is to further quantify the risk of postoperative complications and SSI. At this stage, objective indices such as PNI, CONUT, and GNRI can be incorporated together with clinical factors including age, surgical complexity, expected operative duration, inflammatory status, low muscle mass, or sarcopenia, so as to achieve a more refined secondary stratification of patients (38–40). The advantage of this framework lies in its integration of diagnostic tools and quantitative tools: the former are more useful for capturing the full clinical picture of malnutrition, whereas the latter facilitate standardized and large-scale risk assessment. It should also be noted that the performance of screening tools may vary according to age and clinical setting. For example, in patients with gastrointestinal malignancies, NRS-2002 performs better in identifying GLIM-defined malnutrition among younger patients, whereas older patients may be better evaluated with tools more specifically suited to geriatric characteristics. This also suggests that perioperative nutritional stratification should be tailored to the target population (41).

No single indicator captures the full SSI-risk phenotype. Albumin is influenced by inflammation, capillary permeability, fluid shifts, and catabolism; it should not be interpreted as a pure nutritional marker (42, 43). BMI also cannot distinguish adipose tissue from skeletal muscle and may miss sarcopenia or sarcopenic obesity (44, 45). Accordingly, albumin, BMI, and individual scores should support—not replace—clinical assessment of symptoms, inflammatory burden, functional status, and body composition.

Tool selection should also be adapted to the clinical setting. Older patients often require geriatric-oriented assessment because frailty, appetite loss, multimorbidity, and functional impairment alter risk interpretation (46). Systematic reviews indicate that the performance of nutritional screening tools varies according to the reference standard and target population, and no single tool is uniformly superior across settings (47, 48). A practical SSI-prevention pathway should therefore combine screening, diagnosis, secondary risk quantification, and targeted intervention, with adjustment for age, tumor type, inflammation, and body composition.

## 5 Principles, strengths, and limitations of different nutritional assessment tools

GLIM is more suitable for the diagnosis of malnutrition rather than as a simple screening tool. Its key feature is that it incorporates phenotypic abnormalities, such as weight loss, low BMI, and reduced muscle mass, together with etiologic factors, including reduced intake and inflammation/disease burden, into a single framework. Therefore, it can not only determine whether a patient has malnutrition, but also help explain why such patients are more prone to infection and postoperative complications (49, 50). In surgical populations, the value of GLIM lies mainly in its good correlation with clinical outcomes. A national gastrointestinal surgical registry study showed that malnutrition defined according to the GLIM second-step criteria of weight loss and BMI was associated with an increased incidence of severe complications and a higher 30-day mortality risk after abdominal resection; similarly, in patients with colorectal cancer, preoperative malnutrition defined by GLIM was independently associated with overall postoperative complications (49, 50). In addition, among colorectal cancer patients identified as malnourished by GLIM, the presence of concomitant sarcopenia further increased the risk of postoperative complications, suggesting a close relationship between GLIM and abnormalities in body composition, which is of relevance for understanding the host basis of SSI (51). However, GLIM also has limitations. Its application requires muscle mass assessment, relatively complete medical history data, and information related to inflammation, making clinical implementation relatively complex. Moreover, most current studies have focused on overall postoperative complications rather than SSI alone. Therefore, it is more suitable as an upfront diagnostic tool and should not be used alone for short-term infection risk prediction.

PG-SGA is a clinical nutritional assessment tool specifically designed for patients with cancer. It combines patient self-report with professional evaluation and can more comprehensively reflect recent weight change, reduced food intake, nausea and vomiting, diarrhea, pain, limited activity, and abnormal findings on physical examination. It therefore provides a closer representation of the true nutritional status of cancer patients than tools based solely on laboratory indicators (52). In patients with digestive system malignancies, PG-SGA generally demonstrates strong identification ability. A 2024 hierarchical Bayesian latent class meta-analysis including 33 studies showed that PG-SGA performed better than several commonly used tools in identifying nutritional risk among patients with digestive system tumors; in patients undergoing colorectal cancer resection, malnutrition defined by PG-SGA was also independently associated with postoperative complications (52, 53). In addition, prospective studies have found that PG-SGA scores rise significantly after surgery in patients with gastrointestinal cancer, with the proportion of severe malnutrition increasing from 2.3% preoperatively to 26.3% postoperatively, suggesting that PG-SGA is highly sensitive to changes in perioperative nutritional status (54). Of course, PG-SGA also has limitations. The assessment process is relatively time-consuming and depends heavily on patient-reported symptoms and evaluator experience, so inter-center consistency may be affected by differences in training. At the same time, a comparative study in patients with locally advanced gastric cancer found that PG-SGA was not superior to other objective tools in predicting short-term postoperative events, suggesting that it is more suitable for frontline clinical identification and comprehensive judgment rather than as the optimal tool for short-term risk quantification (55).

PNI is composed of serum albumin and peripheral lymphocyte count and is therefore a simplified composite indicator of nutritional reserve, inflammatory burden, and immune competence (56). Its biological rationale becomes clearer when the two components are considered separately. Albumin is not a pure nutritional marker: a low level may reflect reduced protein reserve, systemic inflammation, increased capillary permeability, fluid redistribution, or enhanced catabolism. These processes may promote edema or effusions and compromise tissue oxygenation and wound repair. Peripheral lymphocyte count provides a complementary immune signal. Lymphopenia reflects impaired cellular immune recovery, weaker T-cell-mediated coordination, and reduced macrophage activation and pathogen clearance. PNI thus compresses two interacting dimensions - protein/inflammation-related tissue vulnerability and cellular immune competence - into a practical index. These mechanisms support its use for risk warning, but they do not prove that changing PNI itself will prevent SSI (17–20, 42, 43).

PNI is simple, inexpensive, and reproducible, making it suitable for retrospective studies and large datasets. Low PNI has been associated with infection after radical gastrectomy and with overall and infectious complications after curative colorectal cancer surgery (57, 58). Low PNI has also been linked to remote infections within 30 days after colorectal surgery, suggesting that it may reflect broader perioperative infectious vulnerability rather than incision risk alone (59). However, PNI is influenced by inflammatory activity, infection, fluid infusion, liver dysfunction, and surgical stress. It should therefore support, not replace, comprehensive nutritional diagnosis and symptom assessment.

Reported PNI thresholds should be interpreted as procedure- and population-specific rather than universal. In colorectal cancer surgery, PNI < =40 was associated with SSI in univariable analysis, whereas studies of major postoperative complications used cut-offs of 39.75 and 44.55 (60–62). In pancreatoduodenectomy, an early postoperative PNI < 40.5 on postoperative day 3 predicted severe complications, although the endpoint was not SSI-specific (63). As a pragmatic clinical reference, a preoperative PNI in the approximate range of 40–45 may justify closer assessment and monitoring, but it should not be treated as a validated universal SSI threshold.

CONUT consists of albumin, peripheral lymphocyte count, and total cholesterol. Compared with PNI, it adds the lipid dimension and is therefore theoretically more helpful in identifying patients who have not yet developed obvious protein abnormalities but already have occult nutritional impairment (64, 65). In gastrointestinal oncologic surgery, CONUT has shown some value in predicting short-term outcomes. A 2023 study suggested that CONUT is an independent risk factor for overall postoperative complications after laparoscopic radical gastrectomy in elderly patients with gastric cancer, and another study in 2025 similarly found that a high preoperative CONUT score was associated with overall postoperative complications after gastric cancer surgery (64, 65). In patients with colorectal cancer, CONUT has also been used to predict postoperative pulmonary complications and surgical outcomes, indicating that its scope of application in perioperative risk assessment is gradually expanding (66). However, the independent predictive value of CONUT for SSI remains inconclusive. On the one hand, it has shown relatively high specificity in some studies and may therefore be suitable for ruling out low-risk patients. On the other hand, the cut-off values differ considerably across surgical procedures, and total cholesterol levels can also be influenced by tumor metabolism, statin use, and systemic inflammation, which limits its generalizability across different settings. Therefore, CONUT is better regarded as a convenient objective supplementary score rather than as a standalone standard for SSI prediction.

GNRI is calculated based on serum albumin and the ratio of current body weight to ideal body weight. It was originally developed as a nutritional risk assessment tool for elderly patients, and is therefore particularly appealing for surgical patients who are older, frail, have a low BMI, or have experienced marked recent weight loss (67, 68). In elderly patients with colorectal cancer, multiple cohort studies have shown that a low GNRI is significantly associated with overall postoperative complications. One study found that GNRI was an independent risk factor for postoperative complications and long-term prognosis in elderly patients with colorectal cancer; another further reported that the incidence of SSI was significantly higher in the low-GNRI group; and in a larger-sample analysis, GNRI <98 was also associated with increased rates of SSI, wound dehiscence, and pneumonia (67–69). Beyond colorectal surgery, a validation cohort in pancreatoduodenectomy also confirmed an association between GNRI and SSI risk, suggesting that its relevance to infectious outcomes is not limited to a single surgical procedure (70). The limitation of GNRI is that its formula depends strongly on body weight and albumin, which may reduce its sensitivity in younger patients, obese patients, and patients with sarcopenic obesity, thereby leading to underestimation of high-risk individuals whose body weight appears acceptable but whose muscle reserve has already markedly declined. Overall, GNRI is more suitable as a simple stratification tool for elderly surgical patients and should not be directly generalized as a universal indicator applicable to all age groups. A comparison of these nutritional assessment tools is shown in Table 1.

**Table 1:** Components, strengths, and limitations of GLIM, PG-SGA, PNI, CONUT, and GNRI.

Tool	Components	Main strengths	Main limitations	References
GLIM	A standardized diagnostic framework combining phenotypic criteria (weight loss, low BMI, reduced muscle mass) with etiologic criteria (reduced intake/assimilation and inflammation or disease burden).	Provides a standardized framework for diagnosing malnutrition and explaining the link between malnutrition, infection susceptibility, and postoperative complications. Strong clinical interpretability in gastrointestinal surgery and colorectal cancer, with associations reported for severe complications, 30-day mortality, and overall postoperative complications. It also shows close coupling with sarcopenia and may help identify patients at particularly high host-risk for SSI.	Implementation is relatively complex because it often requires muscle-mass assessment, detailed history, and inflammatory information. Current evidence is focused more on overall postoperative complications than on SSI as a single endpoint, so it is better suited as an upstream diagnostic framework than as a stand-alone short-term infection prediction tool.	(49–51)
PG-SGA	A cancer-specific clinical assessment tool integrating patient-reported and clinician-assessed information, including recent weight change, dietary intake, gastrointestinal symptoms, pain, functional decline, and physical examination findings.	Captures the real nutritional phenotype of cancer patients more comprehensively than laboratory indicators alone. It has shown good discriminatory ability for nutritional risk in digestive system tumors, is independently associated with postoperative complications after colorectal cancer resection, and is sensitive to perioperative nutritional deterioration.	Assessment is time-consuming and depends on both patient reporting and evaluator experience, which may reduce inter-center consistency. In some comparative studies, it was not superior to objective tools for predicting short-term postoperative events, suggesting greater value for front-end clinical recognition and comprehensive assessment than for rapid short-term risk quantification.	(52–55)
PNI	Calculated from serum albumin and peripheral lymphocyte count; essentially a simplified composite indicator reflecting both nutritional reserve and immune status.	Simple, inexpensive, and highly reproducible, making it particularly suitable for retrospective studies and large datasets. It is widely used in gastrointestinal surgery, and low PNI has been associated with infection after radical gastrectomy, as well as overall and infectious complications after curative colorectal cancer surgery. It may also indicate broader perioperative infectious vulnerability, including remote infections.	Strongly influenced by albumin and lymphocyte values and therefore vulnerable to confounding by inflammation, infection, fluid dilution, liver dysfunction, and perioperative stress. It is best viewed as a rapid objective quantification tool rather than a substitute for a full nutritional diagnosis or symptom-burden assessment.	(56–59)
CONUT	Composed of serum albumin, peripheral lymphocyte count, and total cholesterol; compared with PNI, it adds a lipid dimension and may detect occult nutritional impairment earlier.	A convenient objective score reflecting the inflammation-nutrition interface. It has shown predictive value for overall postoperative complications after laparoscopic-assisted radical gastrectomy in elderly gastric cancer patients and after curative gastric resection. In colorectal cancer, it has also been used to predict postoperative pulmonary complications and surgical outcomes.	Its independent predictive value for SSI remains inconsistent. Cut-off values vary substantially across procedures, limiting generalizability. In addition, total cholesterol can be affected by tumor metabolism, statin therapy, and systemic inflammation, so CONUT is better considered a practical supplementary score than a stand-alone gold standard for SSI prediction.	(64–66)
GNRI	Calculated from serum albumin and the ratio of current body weight to ideal body weight; originally developed to assess nutrition-related risk in older adults.	Particularly attractive for elderly, frail, low-BMI, or recently weight-losing surgical patients. In older colorectal cancer cohorts, low GNRI has been associated with overall postoperative complications, SSI, wound dehiscence, pneumonia, and poorer long-term prognosis. It has also been validated as a predictor of SSI after pancreatoduodenectomy, suggesting that its signal for infectious outcomes is not restricted to a single procedure type.	Because the formula relies heavily on body weight and albumin, it may be less sensitive in younger, obese, or sarcopenic-obese patients and may underestimate risk in patients whose body weight appears acceptable despite marked depletion of muscle reserve. Overall, it is more suitable as a simple stratification tool for older surgical patients than as a universal indicator across all age groups.	(67–70)

GLIM, Global Leadership Initiative on Malnutrition; PG-SGA, Patient-Generated Subjective Global Assessment; PNI, Prognostic Nutritional Index; CONUT, Controlling Nutritional Status; GNRI, Geriatric Nutritional Risk Index; SSI, surgical site infection.

## 6 Integrated evidence on the role of different tools in SSI prevention

The evidence base should be interpreted according to endpoint specificity and study design. Direct SSI evidence is strongest when SSI is a prespecified outcome and definitions are explicit. Evidence based only on overall complications is clinically informative but indirect for SSI prevention. Across the literature, prospective multicenter cohorts and meta-analyses provide stronger support than retrospective single-center studies, yet heterogeneity in surgical populations, cut-off values, and confounding remains substantial. Albumin-based indices are particularly vulnerable to confounding by inflammation, fluid status, liver dysfunction, and acute illness.

Among patients undergoing surgery for gastrointestinal malignancies, existing studies most directly demonstrate the relationship between nutritional risk and infectious complications. Recent data indicate that preoperative nutritional risk is not uncommon in this population. Yang et al. reported that 43.54% of patients with gastrointestinal malignancies had preoperative nutritional risk, and that an NRS-2002 score ≥3 was an independent risk factor for postoperative infection (71). In the field of colorectal cancer surgery, the prospective study by Kwag et al. (72) reached a similar conclusion: patients who screened positive for nutritional risk experienced more postoperative complications, and this risk was not limited to overall complications but also extended to wound infection and anastomosis-related adverse outcomes. From the perspective of objective quantitative indicators, low PNI is closely associated with adverse postoperative outcomes after colorectal cancer surgery. Seretis et al. and Tokunaga et al. respectively confirmed that low PNI was associated with increased overall complications, a higher incidence of severe complications, and poorer prognosis (73, 74). In addition, Sagawa et al. found in a colorectal cancer surgical cohort that, in addition to mGPS, both PNI ≤ 40 and CONUT ≥2 were associated with the occurrence of SSI in univariate analysis, suggesting that nutritional status, inflammatory burden, and host immune function may have additive effects on infection risk (60). Overall, in patients with gastrointestinal malignancies, PG-SGA, NRS, and GLIM are more helpful for identifying the true nutritional phenotype and symptom burden, whereas objective tools such as PNI, GNRI, and CONUT are more suitable for the rapid quantification of complication and SSI risk.

In elderly surgical patients, the importance of nutritional stratification is often even more pronounced. These patients frequently present with frailty, low muscle mass, impaired functional status, and insufficient physiological reserve, and therefore the impact of nutritional abnormalities on postoperative outcomes is more evident. Ścisło et al. (75) found in elderly patients with gastric cancer, pancreatic cancer, and colorectal cancer that malnutrition or risk of malnutrition was closely associated with postoperative complications and prolonged hospital stay, and that SSI accounted for a considerable proportion of these complications. Shen et al. (76) further confirmed that, in elderly patients with colorectal cancer, malnutrition diagnosed by GLIM was not only common but also an independent risk factor for postoperative complications. On the other hand, studies on comprehensive geriatric assessment (CGA) and frailty provide broader support for this view. The prospective cohort study by Kristjansson et al. (77) showed that preoperative CGA helps identify elderly patients with colorectal cancer who are at high risk of severe postoperative complications. The meta-analysis by Xue et al. (78) suggested that CGA components such as comorbidity burden, dependence in activities of daily living, and polypharmacy are all associated with the risk of postoperative complications after gastrointestinal cancer surgery. Moreno-Carmona et al. (79) further showed that frailty significantly increases the risk of postoperative complications and mortality in elderly patients with non-metastatic colon cancer. Therefore, in elderly surgical patients, GLIM and comprehensive geriatric assessment have good complementarity, and objective nutritional indices oriented toward geriatric risk deserve to be incorporated into perioperative SSI risk stratification systems.

Studies from non-gastrointestinal surgical specialties likewise indicate that the relationship between nutritional stratification and infectious complications is not unique to gastrointestinal cancer surgery, but rather represents a clinical phenomenon with broader relevance. In spinal surgery, Ushirozako et al. (80) reported that low preoperative PNI was an independent risk factor for postoperative SSI. Subsequently, two meta-analyses further reinforced this conclusion: Huang et al. (81) found that both PNI and GNRI could be used to predict adverse events after spinal surgery, with low GNRI being associated with an increased risk of SSI; Guo et al. (82) reported that low PNI was significantly associated with increased overall postoperative complications, delirium, and SSI after spinal surgery. In joint surgery, Chen and Chen (83) performed a systematic review and meta-analysis of total joint arthroplasty and found that malnutrition was significantly associated with an increased risk of SSI, with the pooled effect estimate suggesting an approximately 2.6-fold increase in risk. These findings from different specialties suggest that although the infection spectrum and trauma characteristics vary across surgical procedures, inadequate nutritional reserve, increased inflammatory burden, and impaired immune recovery may represent a common host basis. Accordingly, incorporating perioperative nutritional risk stratification into SSI prevention and control should be understood as a broadly applicable cross-specialty strategy rather than merely a special consideration in gastrointestinal surgery.

Although current studies support the clinical value of perioperative nutritional stratification, important limitations remain. Most studies are retrospective and single-center, and many use overall postoperative complications rather than SSI as the primary endpoint. Definitions of SSI, observation windows, cut-off values, and reference standards also vary across studies (84–86). Biochemical surrogates such as albumin and total lymphocyte count can be confounded by inflammation and perioperative stress, limiting causal interpretation (87). Therefore, the current literature supports a multi-tool, stepwise approach, but it does not establish a universally validated SSI-prediction model (Table 2).

**Table 2:** SSI-specific evidence and supportive evidence on nutritional tools across surgical settings.

Surgical setting	Study/population	Tool(s)	Endpoint category	Main findings relevant to SSI/infectious complications	References
Gastrointestinal cancer surgery	Yang, 2024; patients with GI cancers undergoing surgery	NRS-2002	Direct infectious-complication endpoint	Preoperative nutritional risk was common (43.54%); NRS-2002 ≥ 3 was an independent risk factor for postoperative infection.	(71)
Gastrointestinal cancer surgery	Kwag, 2014; colorectal cancer surgery	Nutritional risk screening	Partly direct: morbidity including wound/anastomotic events	Positive nutritional risk screening was independently associated with increased postoperative morbidity and was linked to wound infection and anastomosis-related adverse events.	(72)
Gastrointestinal cancer surgery	Seretis, 2018; elective colorectal cancer resection	Malnutrition status	Indirect supportive evidence: overall postoperative outcomes	Malnutrition was associated with worse postoperative outcomes, supporting the relevance of preoperative nutritional stratification for infectious and overall complications.	(73)
Gastrointestinal cancer surgery	Tokunaga, 2015; colorectal cancer primary tumor resection	PNI	Indirect supportive evidence: severe complications and prognosis	Low PNI predicted severe postoperative complications and poor prognosis, supporting its use as a rapid objective perioperative risk marker.	(74)
Gastrointestinal cancer surgery	Sagawa, 2017; colorectal cancer surgery	mGPS, PNI, CONUT	Direct SSI endpoint	Besides mGPS, PNI ≤ 40 and CONUT ≥2 were associated with SSI in univariable analysis, suggesting additive effects of malnutrition, inflammation, and host immunity on infection risk.	(60)
Elderly GI cancer surgery	Ścisło, 2022; elderly gastric, pancreatic, and colorectal cancer surgery	Nutritional status indicators	Partly direct: complications with SSI proportion reported	Malnutrition or risk of malnutrition was closely associated with postoperative complications and prolonged hospitalization; SSI accounted for a considerable share of complications.	(75)
Elderly GI cancer surgery	Shen, 2023; elderly colorectal cancer surgery	GLIM	Indirect supportive evidence: overall complications and prognosis	GLIM-defined malnutrition was common and independently associated with postoperative complications in older patients undergoing colorectal cancer surgery.	(76)
Elderly GI cancer surgery	Kristjansson, 2010; elderly colorectal cancer elective surgery	CGA	Indirect supportive evidence: severe complications	Preoperative CGA identified older colorectal cancer patients at high risk of severe postoperative complications, supporting geriatric-oriented perioperative stratification.	(77)
Elderly GI cancer surgery	Xue, 2018; meta-analysis in GI cancer patients	CGA components	Indirect supportive evidence: overall complications	Comorbidity burden, ADL dependency, and polypharmacy—core components of CGA—were associated with postoperative complications after GI cancer surgery.	(78)
Elderly GI cancer surgery	Moreno-Carmona, 2024; older patients with non-metastatic colon cancer	Frailty assessment	Indirect supportive evidence: complications and mortality	Frailty significantly increased postoperative complications and mortality risk, reinforcing the value of age-related host vulnerability assessment.	(79)
Spine surgery	Ushirozako, 2021; spine surgery	PNI	Direct SSI endpoint	Low preoperative PNI was an independent risk factor for postoperative SSI after spine surgery.	(80)
Spine surgery	Huang, 2024; meta-analysis of spinal surgeries	PNI, CONUT, GNRI	Direct/combined: adverse events and SSI	Nutritional assessment tools predicted adverse events after spine surgery; low GNRI was associated with increased SSI risk.	(81)
Spine surgery	Guo, 2024; meta-analysis of spine surgery	PNI	Direct/combined: overall complications, delirium, and SSI	Low PNI was significantly associated with increased overall postoperative complications, delirium, and SSI after spine surgery.	(82)
Joint surgery	Chen, 2024; total joint arthroplasty meta-analysis	Malnutrition status	Direct SSI-related endpoint: periprosthetic joint infection and SSI	Malnutrition was significantly associated with periprosthetic joint infection and SSI after total joint arthroplasty; the pooled effect suggested an approximately 2.6-fold higher risk.	(83)

GLIM, Global Leadership Initiative on Malnutrition; PG-SGA, Patient-Generated Subjective Global Assessment; PNI, Prognostic Nutritional Index; CONUT, Controlling Nutritional Status; GNRI, Geriatric Nutritional Risk Index; SSI, surgical site infection, CGA, Comprehensive geriatric assessment.

“Direct” evidence refers to studies reporting SSI or postoperative infection as an explicit endpoint. “Partly direct” evidence includes wound-related or infectious outcomes within broader morbidity analyses. “Indirect supportive” evidence refers to studies reporting overall postoperative complications without an SSI-specific primary endpoint.

## 7 Horizontal comparison of different tools: which is more suitable for SSI risk stratification?

From a methodological perspective, current nutritional tools can be broadly divided into two categories. One category consists of “diagnostic tools,” represented by GLIM and PG-SGA, which focus on determining whether a patient truly has malnutrition and what specific clinical phenotype that malnutrition presents. The other category consists of “scoring tools,” represented by PNI, CONUT, and GNRI, which emphasize rapid risk quantification using a limited number of objective indicators. Recent systematic reviews and meta-analyses have shown that, when PG-SGA is used as the reference standard, GLIM has moderate diagnostic accuracy for malnutrition in patients with cancer, and malnutrition defined by GLIM is associated with an increased risk of postoperative complications; meanwhile, different PG-SGA categories are also associated with poorer survival outcomes and a higher incidence of postoperative complications in patients with cancer (88, 89). This indicates that the main advantage of GLIM and PG-SGA does not lie in providing a simple score, but rather in answering the more fundamental question of whether the patient has clinically meaningful malnutrition. In patients undergoing resection for esophageal squamous cell carcinoma, GLIM and PG-SGA have shown good agreement, and malnutrition identified by either tool has been associated with increased postoperative complications, longer hospital stay, and higher hospitalization costs; however, GLIM showed slightly higher specificity in predicting postoperative complications (90). These findings suggest that the strength of diagnostic tools lies in their ability to link nutritional phenotype, inflammatory background, and clinical outcomes more closely.

When the application scenario is shifted to frontline screening in the preoperative outpatient setting or at the time of hospital admission, the suitability of different tools is not entirely the same. For patients with cancer, especially those with loss of appetite, reduced food intake, gastrointestinal symptoms, or functional limitation, PG-SGA is often more sensitive because it can more systematically capture symptom burden and changes in intake. GLIM, by contrast, is more suitable in settings that require standardized diagnosis and are conducive to research stratification and multicenter comparisons. A 2024 comparative study in patients with hepatocellular carcinoma showed that GLIM, NRS-2002, and PG-SGA can all be used to identify malnutrition, but their diagnostic rates and levels of agreement were not completely consistent, suggesting that in cancer populations the roles of “screening tools” and “diagnostic tools” should be clearly distinguished (91). In contrast, when clinical time is limited and rapid large-scale assessment is needed, GNRI, PNI, and CONUT are easier to apply. A 2025 study in gastric cancer further found that, in identifying GLIM-defined malnutrition, GNRI was superior to PNI and CONUT in terms of specificity, accuracy, and agreement (92). This indicates that objective scoring tools are more suitable for rapid prescreening or for second-step risk quantification, rather than as direct substitutes for clinical nutritional assessment.

If the focus is narrowed further to SSI risk stratification, it is still difficult to conclude that any single tool can serve as the “best predictor” across all surgical specialties. Comparative studies instead suggest that the best-performing tool varies according to the type of surgery and patient population. In perioperative studies of esophageal cancer, GNRI performed better than PNI, NRI, and CONUT in identifying nutritional impairment and predicting infectious complications and major complications, and its results were also more consistent with the ESPEN 2015 diagnostic criteria (93). In contrast, in patients undergoing pancreatoduodenectomy, PNI showed better predictive performance than GNRI and CONUT for both the occurrence and severity of postoperative complications (94). In elderly perioperative patients, a 2023 comparative study showed that GNRI outperformed MNA-SF, NRS-2002, and PNI in predicting short-term outcomes and was more suitable as a rapid screening tool for elderly surgical patients (95). Therefore, a more balanced conclusion would be that GNRI tends to be more informative in older adults and in patients with low body weight or low physiological reserve; PG-SGA has clearer advantages in cancer patients with complex symptom burden; PNI is suitable for coarse stratification and rapid assessment in large samples; and GLIM is more useful for explaining, from a pathophysiological perspective, the relationship between malnutrition and susceptibility to infection.

The most defensible conclusion is complementarity rather than substitution. GLIM and PG-SGA are useful for identifying malnutrition and describing its phenotype, whereas PNI, CONUT, and GNRI are more suitable for rapid objective quantification. However, the evidence does not justify selecting one universal “best” score or treating the proposed tool chain as a validated prediction rule. A stepwise pathway may improve clinical reasoning, but its incremental predictive value over established SSI-risk factors must be tested prospectively.

## 8 Clinical implementation: a practical pathway for perioperative SSI nutritional risk stratification

The following pathway is a hypothesis-generating implementation model rather than a prospectively validated clinical prediction rule. It is intended to organize decision-making within existing perioperative and ERAS workflows while acknowledging that local resources, surgical urgency, and patient characteristics will influence its application.

In routine clinical practice, nutritional risk screening should be performed as early as possible, ideally at the first outpatient surgical evaluation or within 24 h after hospital admission, and should cover all patients scheduled for moderate- to high-risk surgery. For patients with cancer, especially those with gastric cancer, colorectal cancer, and other gastrointestinal malignancies, PG-SGA is more appropriate as the preferred tool for evaluating symptom burden, reduced food intake, and weight change. In contrast, for patients undergoing general surgery or routine abdominal procedures, NRS-2002 may be used first for rapid screening, with those who screen positive subsequently entering the GLIM diagnostic pathway. Previous studies have shown that higher NRS-2002 scores are closely associated with an increased risk of postoperative complications; in patients with gastrointestinal malignancies, both nutritional risk identified by NRS-2002 and malnutrition diagnosed by GLIM are independent predictors of postoperative complications (96, 97). In addition, initial outpatient screening should not be limited to body weight and albumin alone, but should also identify marked recent weight loss, restricted food intake, geriatric frailty, and low muscle mass. This is because when nutritional depletion, frailty, and sarcopenia coexist, their predictive value for postoperative sepsis and poor recovery is significantly strengthened (98).

For patients who screen positive, the next step should be entry into first-level stratification. The goal at this stage is not merely to determine whether “nutrition is poor,” but rather to further clarify which malnutrition phenotype the patient belongs to. At this stage, GLIM is more suitable for standardized diagnosis because it requires simultaneous consideration of phenotypic criteria, such as weight loss, low BMI, and reduced muscle mass, together with etiologic criteria, including reduced intake/malabsorption or inflammation/disease burden. PG-SGA, by contrast, is better suited to reflecting the clinical complexity of patients with cancer, such as anorexia, fatigue, nausea, functional decline, and a tendency toward cachexia. Studies have shown that malnutrition diagnosed by GLIM is associated with an increased incidence of pulmonary complications and a higher 90-day mortality risk after major abdominal surgery in patients with cancer; among patients with gastric cancer, more severe GLIM stages are also associated with higher rates of postoperative complications, longer hospital stay, and greater hospitalization costs (99, 100). On the other hand, PG-SGA not only reflects symptom burden and intake problems, but is also closely associated with sarcopenia. In patients with gastrointestinal malignancies, a PG-SGA score ≥7 is significantly associated with an increased risk of sarcopenia. Therefore, during first-level stratification, recent weight change, BMI, reduced intake, inflammatory background, and abnormalities in body composition should be documented simultaneously, so as to avoid missing concealed high-risk patients whose BMI appears normal but who already have low muscle mass (101).

Once nutritional abnormalities have been confirmed, patients should proceed to second-level stratification, the purpose of which is to more precisely quantify susceptibility to SSI and, on this basis, classify patients into four categories: low risk, moderate risk, high risk, and very high risk. At this stage, PNI, CONUT, and GNRI may be used as objective weighted indicators, while traditional SSI risk factors should also be incorporated, such as age, diabetes, anemia, obesity or low muscle mass, tumor stage, degree of contamination, surgical complexity, expected operative duration, and the potential need for stoma creation. Studies in colorectal surgery have shown that diabetes, obesity, wound contamination, and prolonged operative time are all independent risk factors for SSI, while low albumin and low hematocrit also often indicate higher susceptibility to infection (102). In patients with gastric cancer, preoperative sarcopenia itself has likewise been confirmed to be an independent predictor of severe postoperative complications (103). For elderly or low-body-weight patients, GNRI generally performs more consistently; in geriatric spinal surgery populations, GNRI has shown better predictive accuracy than PNI and CONUT for perioperative adverse events, suggesting that GNRI may be preferentially incorporated into the second-step quantification framework in elderly surgical patients (104). Therefore, a more practical second-level stratification model should be based on the combination of objective nutritional indices + traditional SSI risk factors + body composition/frailty information, rather than relying on any single score.

A validated quantitative scoring system combining nutritional indices with conventional SSI risk factors is currently not available. It would be premature to assign fixed points - for example, to PNI < 40, age >70 years, diabetes, or operative time >3 h - because effect estimates and cut-offs vary across procedures, populations, and endpoint definitions. Future derivation studies should prespecify candidate variables, construct weighted models from multivariable effect estimates, and perform internal and external validation before any score is used to guide perioperative care.

After completion of the two-level stratification process, perioperative intervention strategies may be further mapped to a four-tiered pathway. Low-risk patients may be managed according to the standard ERAS pathway, with a focus on preoperative education, avoidance of unnecessary fasting, and early resumption of oral intake within 24 h after surgery, since early oral feeding has been shown to be safe and beneficial for recovery in both colorectal and gastric cancer surgery (11, 105–109). Moderate-risk patients should receive early consultation by a dietitian, begin oral nutritional supplementation, increase protein intake, and simultaneously optimize perioperative metabolic control. Updated Cochrane evidence suggests that, in patients with weight loss or malnutrition, standard oral nutritional supplementation can reduce the risk of infection to some extent; meta-analyses in patients with gastrointestinal malignancies have also shown that preoperative oral nutritional supplementation helps reduce infectious complications and improve nutritional and immune status (11, 106). High-risk patients, if surgery can be safely delayed, should be given as much as possible a 7–14-day window for nutritional prehabilitation, with enteral nutrition or standardized nutritional supplementation added when necessary. Classical randomized controlled trials have shown that preoperative specialized nutritional supplementation can improve surgical outcomes in patients with gastrointestinal cancer, and that the effect of multimodal prehabilitation is also influenced by baseline nutritional status (105, 107). Very high-risk patients are more appropriately managed through a multidisciplinary pathway involving surgery, anesthesia, nutrition, infection, and rehabilitation teams, with surgery deferred when necessary until more intensive nutritional support and metabolic optimization have been completed preoperatively. Existing studies have shown that, in elderly patients with abdominal malignancies, nutritional prehabilitation can reduce the incidence of infection and shorten hospital stay (106).

In practice, the pathway can be simplified according to patient type and available resources. For patients with gastrointestinal cancer or substantial symptom burden, PG-SGA should be prioritized, followed by GLIM diagnosis and secondary quantification with PNI, CONUT, or GNRI. For older or frail patients, GNRI should be interpreted together with frailty, functional status, and body-composition assessment. For general abdominal surgery, NRS-2002 can serve as a rapid entry screen, with GLIM applied when screening is positive. In resource-limited settings, a pragmatic minimum pathway is NRS-2002 plus routine laboratory indices, with dietitian referral and expanded assessment for patients who screen positive or have major clinical risk factors.

**Figure 2:**
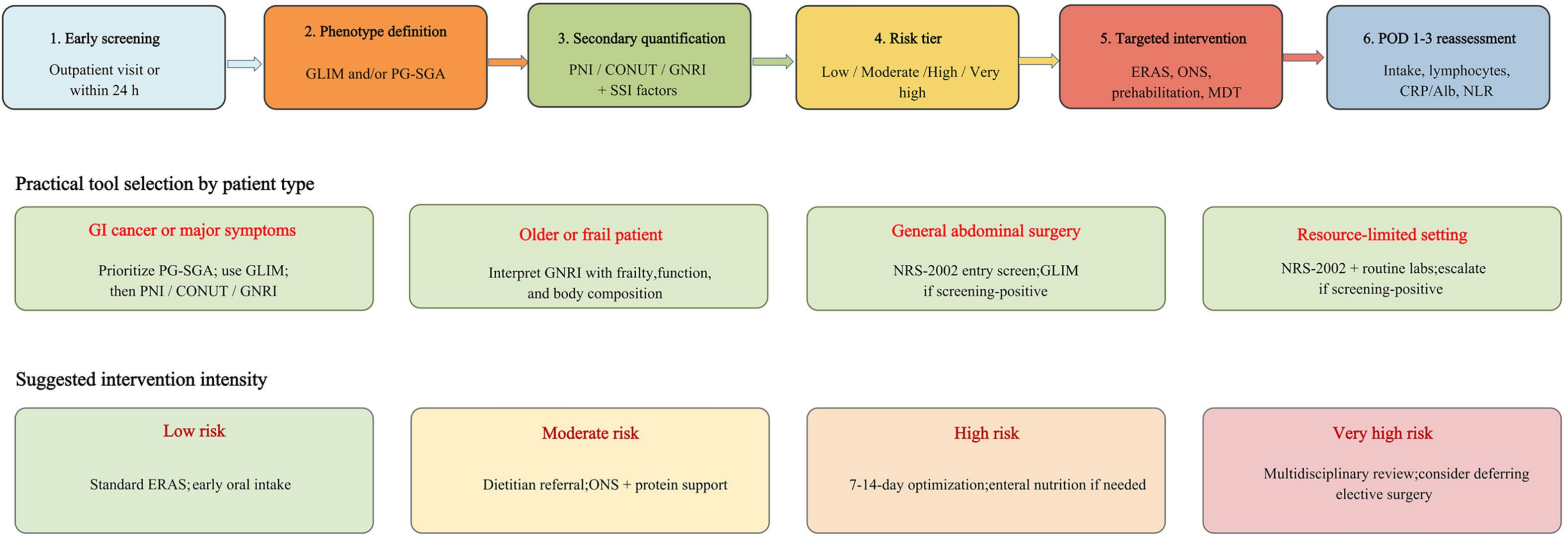
Hypothesis-generating flowchart of perioperative SSI nutritional risk stratification and precision intervention.

In addition to preoperative stratification, postoperative reassessment on postoperative days 1–3 should also be incorporated into the SSI prevention pathway, because this period is often the earliest critical window in which “failure of nutritional recovery” and “loss of inflammatory control” become apparent. In clinical practice, the nadir peripheral lymphocyte count, degree of albumin decline, recovery of oral intake, and combined inflammatory-nutritional indicators such as CRP and the CRP/Alb ratio may be dynamically monitored and regarded as warning signals preceding infection. Studies in colorectal surgery have shown that the postoperative CRP/Alb ratio on POD3 has greater diagnostic accuracy than CRP alone for both overall complications and SSI, while poGPS on POD3–4 also shows a clear gradient relationship with the incidence and severity of infectious complications (110, 111). In addition, studies from other surgical specialties also support this concept of “dynamic immune recovery”: in lumbar spine surgery, NLR on postoperative days 4 and 7 can be used to predict SSI; in pediatric cardiac surgery, a lower lymphocyte nadir during the first 2 postoperative days is independently associated with an increased risk of infection within 30 days (112, 113). Therefore, postoperative reassessment should not regard nutrition merely as a background component of supportive care, but should instead consider poor early recovery of oral intake, inadequate lymphocyte recovery, and persistently abnormal inflammatory-nutritional ratios as important signals of secondary high risk, and should prompt timely escalation of monitoring and intervention accordingly (114, 115) (Figure 2 and Table 3).

**Table 3:** Suggested SSI-prevention interventions within the proposed nutritional risk-stratification pathway.

Stage/risk tier	Applicable patients	Recommended tools/pathway	Suggested SSI-prevention interventions	References
Universal preoperative screening	All patients scheduled for intermediate- or high-risk surgery; ideally at the first surgical outpatient visit or within 24 h after admission	For cancer patients—especially gastric, colorectal, and other gastrointestinal malignancies—prioritize PG-SGA to capture symptom burden, reduced intake, and recent weight loss. For general/abdominal surgical patients, start with NRS-2002 and proceed to GLIM if screening-positive. Actively identify marked recent weight loss, restricted intake, frailty, and low muscle mass.	Move screening upstream; do not rely solely on body weight or albumin. Early identification of nutritional depletion, frailty, and sarcopenia helps flag patients at higher risk of infection, sepsis, and poor recovery.	(96–98)
Level 1 stratification (phenotype clarification)	Patients with positive initial screening or suspected nutritional abnormality	Use GLIM for standardized diagnosis by combining phenotypic criteria (weight loss, low BMI, reduced muscle mass) and etiologic criteria (reduced intake/absorption or inflammation/disease burden). Use PG-SGA to characterize anorexia, fatigue, nausea, functional decline, and cachexia tendency. Record recent weight change, BMI, intake decline, inflammatory background, and body-composition abnormalities.	Identify the specific malnutrition phenotype rather than making a simple yes/no judgment. This step is particularly important for detecting occult high-risk patients with preserved BMI but low muscle reserve.	(99–101)
Level 2 stratification (SSI susceptibility quantification)	Patients with confirmed nutritional abnormality after Level 1 assessment	Use objective indices such as PNI, CONUT, and GNRI, and combine them with conventional SSI risk factors including age, diabetes, anemia, obesity or low muscle mass, tumor stage, wound contamination, operative complexity, expected duration, and possible stoma creation. In older or low-body-weight patients, GNRI may be prioritized.	Adopt a composite model of nutritional indices + conventional SSI factors + body-composition/frailty information, rather than relying on any single score.	(102–104)
Low risk	No definite malnutrition; low SSI burden after integrated stratification	Standard ERAS-based perioperative care	Provide preoperative education, avoid unnecessary fasting, and resume oral intake early (ideally within 24 h after surgery) when feasible. Early oral feeding has been shown to be safe and recovery-promoting after colorectal and gastric cancer surgery.	(105, 110, 111)
Moderate risk	Mild nutritional impairment or moderate SSI susceptibility	Early dietitian consultation + oral nutritional supplementation (ONS) + protein reinforcement + metabolic optimization	Start ONS as early as possible, reinforce protein intake, and optimize perioperative metabolic control. Evidence suggests that standard ONS can modestly reduce infection risk in patients with weight loss or malnutrition, and preoperative ONS may decrease infectious complications while improving nutritional and immune status in gastrointestinal cancer surgery.	(11, 106)
High risk	Marked nutritional impairment and/or high predicted SSI risk, but surgery can still be postponed	Nutritional prehabilitation window (preferably 7–14 days) + enteral nutrition when needed + standard nutritional supplementation	When oncologically and logistically feasible, delay surgery briefly to create a 7–14-day optimization window. Preoperative specialized nutritional support may improve gastrointestinal cancer surgery outcomes, and the effect of multimodal prehabilitation is modified by baseline nutritional status.	(105, 108, 109)
Very high risk	Severe malnutrition, frailty, sarcopenia, major metabolic derangement, or expected highly complex surgery	Multidisciplinary perioperative pathway involving surgery, anesthesia, nutrition, infection control, and rehabilitation; consider more intensive nutritional support and metabolic optimization before surgery	Escalate to multidisciplinary management rather than proceeding with routine perioperative care. In elderly patients undergoing gastrointestinal cancer surgery, preoperative oral nutritional support/prehabilitation has been associated with reduced complications and shorter hospital stay.	(106, 107)
Postoperative re-stratification (POD 1–3)	All patients, especially those with preoperative nutritional risk or delayed recovery	Dynamically assess the nadir lymphocyte count, magnitude of albumin decline, recovery of oral intake, and combined inflammation–nutrition markers such as CRP and the CRP/albumin ratio. Consider postoperative inflammation scores where available.	Treat poor early intake recovery, poor lymphocyte recovery, and persistently abnormal inflammation–nutrition ratios as warning signs of secondary high risk. POD3 CRP/Alb and postoperative inflammation scores are associated with overall and infectious complications after colorectal surgery; postoperative NLR and lymphocyte nadir also support early infection prediction in other surgical settings.	(112–115)

GLIM, Global Leadership Initiative on Malnutrition; PG-SGA, Patient-Generated Subjective Global Assessment; PNI, Prognostic Nutritional Index; CONUT, Controlling Nutritional Status; GNRI, Geriatric Nutritional Risk Index; SSI, surgical site infection.

This pathway is a hypothesis-generating framework. Risk tiers and intervention intensity should be adapted to surgical urgency, local resources, and multidisciplinary judgment until prospective validation is available.

Static nutritional markers should also be interpreted cautiously. A single preoperative PNI measurement cannot capture rapid postoperative changes caused by surgical stress, fluid redistribution, inflammatory activation, and delayed recovery of oral intake. Future prospective studies should measure PNI at baseline, postoperative days 1–3, day 7, and day 14, and relate both absolute values and delta-PNI trajectories to the timing and type of SSI onset. These studies should distinguish superficial incisional, deep incisional, and organ/space SSI and use time-to-event methods to test whether persistent PNI suppression or failure of recovery adds predictive value beyond baseline PNI and established SSI-risk factors. Evidence that postoperative day-3 PNI predicts severe complications after pancreatoduodenectomy, together with dynamic CRP/albumin ratio, postoperative inflammation score, NLR, and lymphocyte-nadir data, supports this research direction but does not yet validate a PNI-trajectory algorithm for SSI (63, 110–115).

The proposed pathway has not yet been prospectively validated. Future studies should compare the stepwise model with usual care and with single-tool strategies, assess calibration and discrimination for standardized SSI endpoints, and evaluate whether risk-guided nutritional intervention reduces infection rates, length of stay, and resource use. External validation across cancer surgery, geriatric surgery, and general abdominal surgery will be necessary before the pathway can be treated as a standardized algorithm. An additional priority is the development of dynamic models that combine baseline phenotype with serial PNI or related trajectories and explicitly align biomarker changes with the timing of SSI onset.

## 9 Controversies and unresolved issues

At present, one of the primary problems in using perioperative nutritional tools for SSI risk stratification is the lack of a unified standard for cut-off values. Even among patients undergoing the same type of colorectal cancer surgery, the PNI thresholds reported in different studies vary substantially. Guldogan et al. (61) reported an optimal cut-off of 39.75 for predicting major complications, whereas Cao et al. (62) proposed a threshold of 44.55 in a cohort undergoing curative laparoscopic surgery. Similar inconsistencies are also seen with CONUT and GNRI. Ahiko et al. (116) used CONUT ≥4 to predict short-term complications in elderly patients with colorectal cancer, whereas Liao et al. (117) used GNRI <98 to identify patients at high risk for complications and SSI. A systematic review further pointed out that the optimal GNRI threshold remains inconsistent across studies and still requires clarification through larger-sample investigations (118). These discrepancies in threshold values mean that the comparability of the same tool across different surgical procedures, age groups, and study endpoints is limited, which also affects its broader application as a unified standard for SSI stratification.

Another important issue is the substantial heterogeneity of the SSI endpoint itself. In existing studies, some define outcomes as overall infectious complications, some include only superficial SSI, deep SSI, or organ/space SSI, while others also incorporate pulmonary infections, remote infections, or even infections occurring after readmission into the analysis. As a result, although these studies appear to be discussing “infection risk,” the actual endpoints are not entirely consistent. In spinal surgery research, Nota et al. (119) pointed out that the incidence of SSI changes significantly depending on the definition of infection used. Christensen et al. (120) also showed that, even when focusing on SSI, the two surveillance systems, NHSN and NSQIP, differ in their definitional logic and case-capture methods, and these differences may even affect benchmark comparisons between hospitals. Therefore, the differences among studies regarding which tool—PNI, CONUT, GNRI, or GLIM/PG-SGA—is “better” may not be entirely attributable to the tools themselves, but may instead partly reflect inconsistencies in outcome definitions, observation windows, and case-classification standards.

A third unresolved issue is the dual nature of albumin within these tools. For a long time, albumin has been used as a nutritional marker, but a low albumin level does not necessarily indicate simple inadequate nutritional intake. The systematic review by Lee et al. (121) showed that, in individuals without disease but with marked caloric restriction, albumin and prealbumin generally do not decline significantly until extreme starvation occurs; by contrast, under conditions of injury or disease, these markers may decline rapidly even before nutritional intake has changed substantially, indicating that they are intrinsically closer to negative acute-phase proteins. Gradel’s (122) literature review likewise pointed out that many prognostic indices based on albumin actually contain two layers of information simultaneously: “nutrition” and “inflammation.” This means that scoring tools such as PNI, CONUT, and GNRI that include albumin are indeed clinically valuable, but what they reflect is not purely nutritional reserve; rather, they more likely represent the combined effects of malnutrition, inflammatory burden, and changes in fluid status. For this very reason, such indices have both strengths and limitations when interpreting SSI risk: they are useful, but they lack complete specificity.

A fourth controversy is that the agreement between different tools is not stable, suggesting that they may not be identifying the same type of nutritional abnormality. In 2025, Kong et al. (123) found in patients with esophageal cancer that the agreement of PG-SGA with GLIM and with NRS2002-GLIM was weak, with a kappa value of only about 0.38, and the prevalence of malnutrition differed substantially depending on the method used. However, in the same year, Wang et al. reported in patients with pancreatic cancer that the agreement between GLIM and PG-SGA reached *κ* = 0.71, representing a relatively high level of consistency (124). This indicates that the degree of agreement between PG-SGA and GLIM is influenced by multiple factors, including tumor type, screening entry point, symptom burden, methods of body composition assessment, and criteria for severity classification. In other words, these two tools are not simply interchangeable, but are more likely oriented toward identifying different dimensions of nutritional abnormalities: PG-SGA places greater emphasis on clinical symptoms and functional status, whereas GLIM focuses more on standardized diagnosis and the integration of inflammation with phenotypic features.

A fifth unresolved issue is temporal validity. PNI, CONUT, GNRI, and albumin-based measures are often treated as static baseline variables, but surgery can rapidly change albumin concentration, lymphocyte count, fluid balance, and inflammatory activity. A low postoperative value may represent pre-existing vulnerability, the magnitude of surgical stress, early complications, or a combination of these factors. Prospective trajectory studies are therefore needed to determine whether recovery patterns from the preoperative period to postoperative day 14 improve SSI prediction and whether different trajectories precede different SSI phenotypes.

Finally, the field lacks intervention trials that enroll patients according to predefined nutritional-risk strata and use standardized SSI endpoints. Although preoperative nutritional support and prehabilitation are increasingly studied, it remains uncertain whether the same intervention intensity is appropriate for all patients or whether benefit is concentrated in selected high-risk phenotypes (10, 125). This gap is central: nutritional stratification should be judged not only by its association with outcomes, but also by whether it improves treatment allocation and reduces SSI in prospective comparative studies.

## 10 Conclusion

In summary, perioperative malnutrition is an SSI-susceptible host state shaped by inadequate nutritional reserve, inflammation, delayed immune recovery, low muscle mass, and impaired wound repair. GLIM and PG-SGA are useful for identifying clinically meaningful malnutrition phenotypes, whereas PNI, CONUT, and GNRI provide rapid objective quantification. Current evidence supports a complementary, stepwise approach rather than reliance on any single tool. However, SSI-specific evidence remains less robust than evidence for overall postoperative complications, and albumin-based indices are susceptible to confounding. The proposed pathway should therefore be viewed as a hypothesis-generating framework for integration into ERAS and perioperative workflows. Prospective multicenter validation using standardized SSI definitions is required before routine adoption.
